# *In-Operando* FTIR Spectroscopy Study on Rapid Polysulfide Binding via Caffeine in Lithium–Sulfur Batteries

**DOI:** 10.1021/acs.jpclett.4c02789

**Published:** 2024-12-02

**Authors:** Taber Yim, Rhyz Pereira, Jantakan Nedsaengtip, Aaron T. Fafarman, Vibha Kalra

**Affiliations:** † Department of Chemical and Biological Engineering, Drexel University, Philadelphia, Pennsylvania 19104, United States; ‡ Smith School of Chemical and Biomolecular Engineering, Cornell University, Ithaca, New York 14853, United States

## Abstract

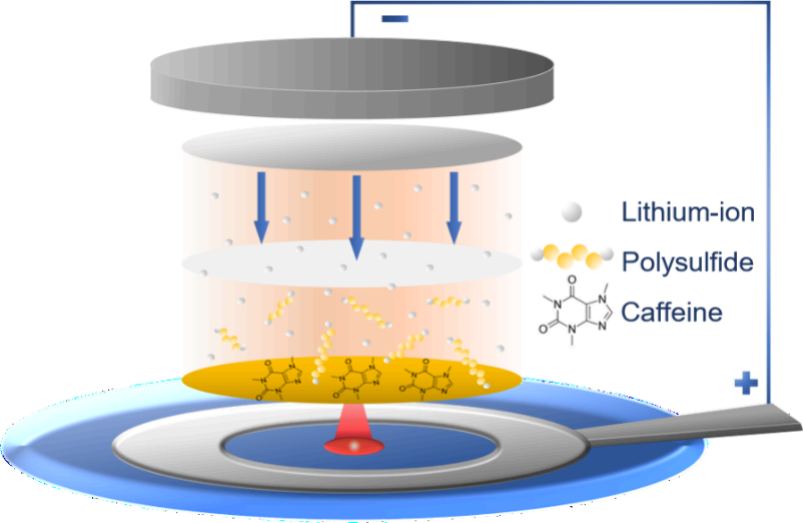

Lithium–sulfur batteries are limited by the high mobility of polysulfides in the electrolyte, which allows them to migrate from the cathode to the lithium anode. This is known as polysulfide shuttling and simultaneously diminishes the active material and poisons the anode. Various cathode additives have been shown empirically to mitigate this problem, although the mechanism is not often ascertained experimentally. Herein, we demonstrate for the first time that the small molecule additive caffeine reduces the polysulfide shuttling current in a lithium–sulfur battery and decreases the capacity fade in galvanostatic cycling experiments. Using *in-operando* Fourier transform infrared (FTIR) spectroscopy, we identify reversible shifts in the carbonyl stretching frequencies of caffeine that are strongly correlated with the onset of polysulfide formation during both discharging and charging. These spectroscopic shifts are consistent with a polar–polar interaction between polysulfides and the carbonyl groups of caffeine, leading to the observed decrease in polysulfide mobility.

Lithium-ion batteries are a mature technology with energy densities around 150–250 Wh kg^–1^. They are widely implemented, but weight and cost reductions are becoming limited. In contrast, lithium–sulfur batteries (LSBs) are a promising next generation battery chemistry with low costs, low environmental impact, and a theoretical energy density around 2,600 Wh kg^–1^, much higher than LIBs.^1−5^ However, LSBs are hindered by the polysulfide shuttling effect, whereby intermediate polysulfide species can dissolve in the liquid electrolyte and shuttle from the cathode to the lithium anode.^6^ There, polysulfides rapidly reduce, poisoning the anode and decreasing active material, ultimately leading to capacity fade.^7^

To combat this shuttling effect, various strategies have been used. Approaches from the electrolyte include electrolyte additives, inert cosolvents, and phase separating electrolytes.^8−10^ A less commonly used class of additives are molecules that create complexes with polysulfides that are too large to diffuse through the conventional separator pores, such as an organic oligoaniline.^11^ Other methods focus on the cathode structure to physically encapsulate sulfur and prevent it from leaving the cathode.^12−14^ In a recent discovery, Pai et al. modified the sulfur active material itself, synthesizing a γ-monoclinic sulfur that could undergo a direct, solid–solid conversion from elemental sulfur to Li_2_S.^15^ In a cathode additive approach, Cardoza et al. studied 1-dimensional titania lepidocrocite-based nanofilaments that could anchor polysulfides both through polar–polar and Lewis acid–base interactions.^16^ Natural materials like gelatin, zein proteins,^17^ amino acids, and soy protein^18^ have also seen success due to their polar functional groups. Other methods include improving necessary battery components such as functionalized cathode binders^19,20^ and separators.^21^

To fundamentally study the interactions of these materials with polysulfides, *in-operando* techniques are greatly beneficial. *In-operando* Fourier transform infrared (FTIR) spectroscopy is a powerful technique for studying bond signatures and changes in materials and does not require an X-ray source. However, few studies exist in the literature due to stringent requirements of the system including simulating mechanical coin cell crimping pressure, hermetic sealing, demonstration of proper electrochemical performance in the model system, and coupling of optical and electrochemical signals. Of the few studies leveraging this technique for lithium–sulfur batteries, most have investigated polysulfide speciation.^22−25^ Some works also investigated electrolyte decomposition.^26,27^ Beyond these, *in-operando* FTIR studies of LSBs in real time are limited. Our study demonstrates the utility of this technique beyond speciation to tackle the mechanistic understanding of polysulfide interaction with the molecular additive caffeine.

Motivated by the natural material approach and the use of polar functional groups to bind polysulfides, we studied caffeine as a cathode additive in LSBs. Caffeine, a type of methylxanthine, has never been studied before in the context of LSBs. In this work, we first studied polysulfide shuttle inhibition through electrochemical measurements, including a shuttle current test, and saw evidence of reduced polysulfide shuttling when caffeine was present. Furthermore, we saw capacity retention improvements in the galvanostatic cycling of full LSB cells. To fundamentally investigate these phenomena, we used *in-operando* FTIR to study caffeine. By tracking spectroscopic shifts of the carbonyl peaks on caffeine, we observed a reversible lithium polysulfide binding effect that reduced shuttling. Additionally, the carbonyl peak shifts corresponded directly with the appearance of polysulfides in the battery during both charging and discharging, illustrating the rapid binding response of caffeine. Finally, we evaluated the unique shifting behavior of each carbonyl group to propose the preferred lithium polysulfide binding site on caffeine.

To first study the effect of caffeine in a LSB, it was incorporated as a solid powder additive into the cathode (Figure S1, SEM images), with the carbonyl groups of caffeine remaining unchanged during the fabrication process, as seen by FTIR and X-ray photoelectron spectroscopy (XPS) measurements (Figures S2 and S3). Caffeine was added with respect to the mass of sulfur, keeping sulfur loading constant. Herein, cathodes with caffeine will be referred to by their sulfur:caffeine ratio. For example, a cathode with a 4:1 ratio of sulfur:caffeine will be referred to as a 4:1 cathode. Figure 1a shows a shuttle current test for a reference sulfur cathode without caffeine, a 10:1 cathode, and a 4:1 cathode. Achieving higher caffeine mass loadings in the cathode with our fabrication process was not possible due to cathode cracking. In the cell, polysulfides shuttle due to concentration gradients. This test measures the applied current necessary to maintain an open circuit voltage (OCV) in the cell to combat polysulfide shuttling. A lower current indicates less shuttling.^28^ The reference cathode shows a current of 7.5 μA at 4 h, whereas the 4:1 cathode shows a current of 2.3 μA, a 67% reduction. The 10:1 cathode shows a current of 6.0 μA which is a small improvement from the reference but much less than the 4:1 cathode. UV–vis measurements showing a decrease in polysulfide peak intensity after exposure to caffeine support these findings (Figure S4).

**Figure 1:**
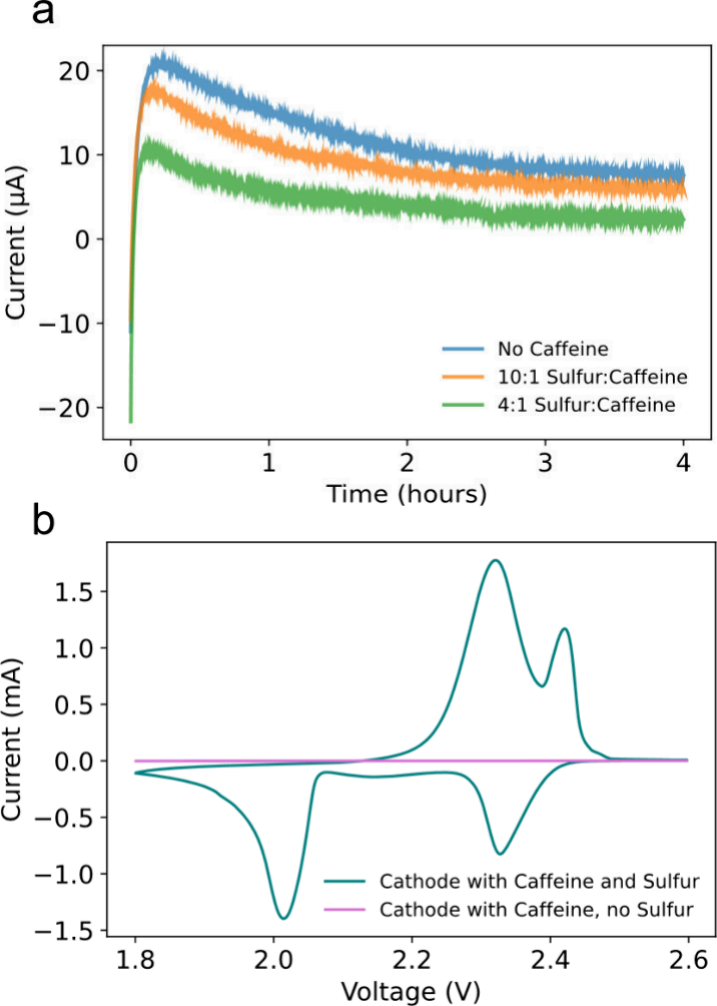
(a) Shuttle current test for sulfur cathodes with varying ratios of sulfur:caffeine. (b) CV comparison of cathodes with caffeine only and caffeine with sulfur.

Figure 1b shows CVs of a conventional cathode with only caffeine (no sulfur) as a reference and a cathode with both caffeine and sulfur. The CV profile for the caffeine only cathode shows no features, indicating an absence of redox activity. This demonstrates that caffeine itself is not electrochemically active in the voltage window of 2.6 to 1.8 V and stable with other LSB components. The CV with caffeine and sulfur exhibits the expected two-peak behavior of the Li–S chemistry.

Figure 2a shows a galvanostatic cycling plot comparing Li–S coin cells with various ratios of caffeine in the cathode cycled at a C/2 rate. The 10:1 cell shows a minimal difference in performance compared to the cell with no caffeine, supporting the findings from the shuttle current test. This galvanostatic test reflects more factors than shuttle current alone, and evidently the small improvements in shuttle current at lower caffeine mass-fraction are insufficient to differentiate it from the control by this measure. In contrast, the 4:1 cell showed a clear improvement. At 300 cycles, the 4:1 cell provides a capacity of 500 mAh g^–1^, compared to 354 mAh g^–1^ in the conventional cell, a 40% improvement (comparison with other works in Table S1). We hypothesize that the increased capacity could be due to higher sulfur utilization, enabled by lithium polysulfide binding via caffeine molecules. Still, due to the severe polysulfide shuttling issue, capacity fade can be seen in all cells. It is especially prominent in the earlier cycles and decreases once the lithium anode is more passivated. The caffeine additive also mitigated this effect. However, due to volume expansion in the cell and parasitic side reactions, passivation layers can be fractured and capacity fade persists throughout cycling.^29,30^Figure 2b shows the voltage curve corresponding to the fifth cycle of galvanostatic cycling and Figure 2c from the 250th cycle. Both figures show that the 2.3 V plateau remains larger throughout cycling in the 4:1 cell. Since the 2.3 V plateau is where polysulfides are first formed in the cell, it suggests that the caffeine is binding long-chain lithium polysulfides and retaining active material at the cathode. This anchoring effect is supported by the shuttle current test. The 4:1 cell shows a slightly greater overpotential at the 2.1 V plateau that grows larger from the 5th to the 250th cycle. Still, the 4:1 cell has a longer 2.1 V plateau that reflects higher sulfur utilization and greater capacity. Our hypothesis for the larger overpotential at the 2.1 V plateau is that slightly more energy is required for polysulfide conversion and deposition at the cathode. This may be caused by the reduction process occurring in more localized areas near caffeine, due to lithium polysulfides interacting with it. This may in turn create localized areas of insulating species on that cathode that could cause an increase in overpotential.^31,32^ This hypothesis is supported by Li_2_S nucleation measurements and electrochemical impedance spectroscopy (EIS) (Figures S5 and S6). Nevertheless, the polysulfide binding phenomenon displayed by caffeine provides an overall benefit, especially at lower C-rates (Figure S7). Furthermore, the conversion of polysulfides back to sulfur does not appear to be hindered, as shown by the similarly shaped charging voltage profiles (Figure 2b,c).

**Figure 2:**
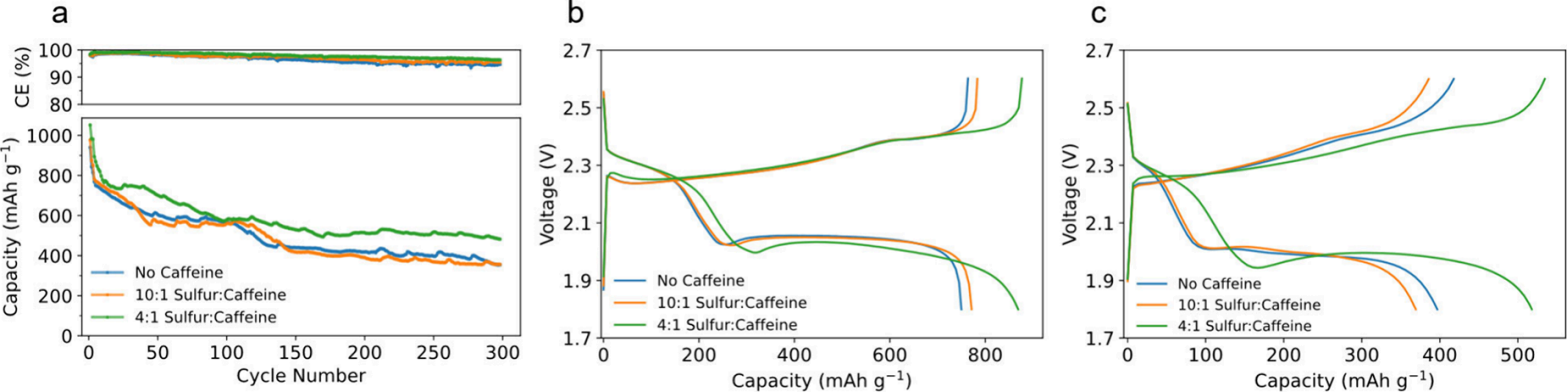
(a) Galvanostatic cycling comparison of lithium–sulfur coin cells with and without caffeine incorporated into the cathode. Corresponding voltage curves from the (b) 5th and (c) 250th cycle.

To fundamentally understand the phenomenon seen in the electrochemical analysis, we use *in-operando* FTIR to observe spectral changes in the battery as it cycles. Scheme 1 illustrates the physical setup of the *in-operando* FTIR cell. The cathode for this system was created by casting a 4:1 slurry on a Celgard separator, eliminating the need for an aluminum current collector that would otherwise block the beam. To identify noteworthy peaks, we established baseline caffeine peak positions with the raw materials. In the spectra for pure caffeine powder (Figure S8a), the peak at 1,647 cm^–1^ can be assigned to the carbonyl stretching mode primarily localized at position C6 (Figure 3a) and the peak at 1,694 cm^–1^ is likewise assigned to position C2.^33,34^ These peaks are slightly shifted when caffeine is solvated in ether but remain clearly distinguishable. Furthermore, there is no signal from the solvent in this region that masks the carbonyl peaks. Amines are another polar group of interest for their ability to interact with polysulfides, as suggested by DFT calculations.^35−39^ In our observed spectra (Figure S8b), the characteristic tertiary amine peaks of caffeine at 1,025 and 975 cm^–1^ are masked by the signal from the ether;^40^ therefore, we cannot say whether there are also changes in this region.

**Scheme 1:**
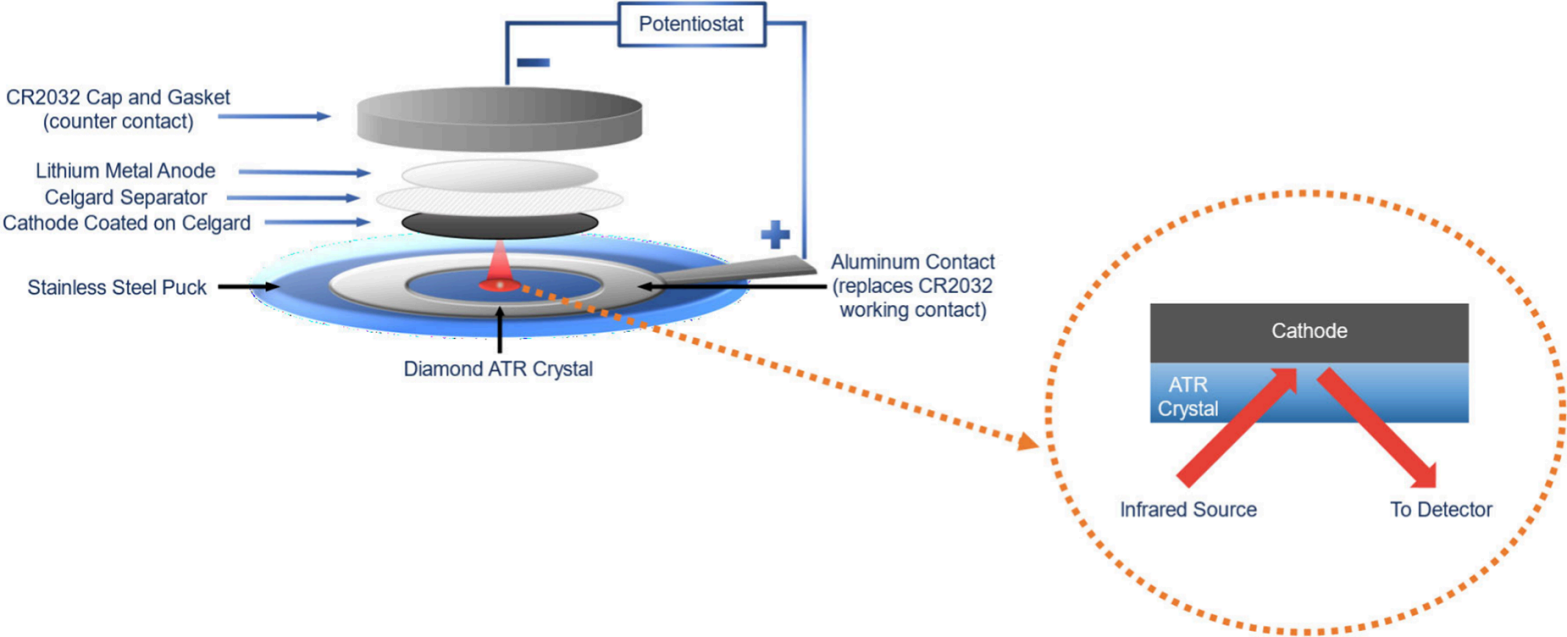
Schematic of the *in-Operando* FTIR Coin Cell Setup Featuring an ATR Diamond Crystal

**Figure 3:**
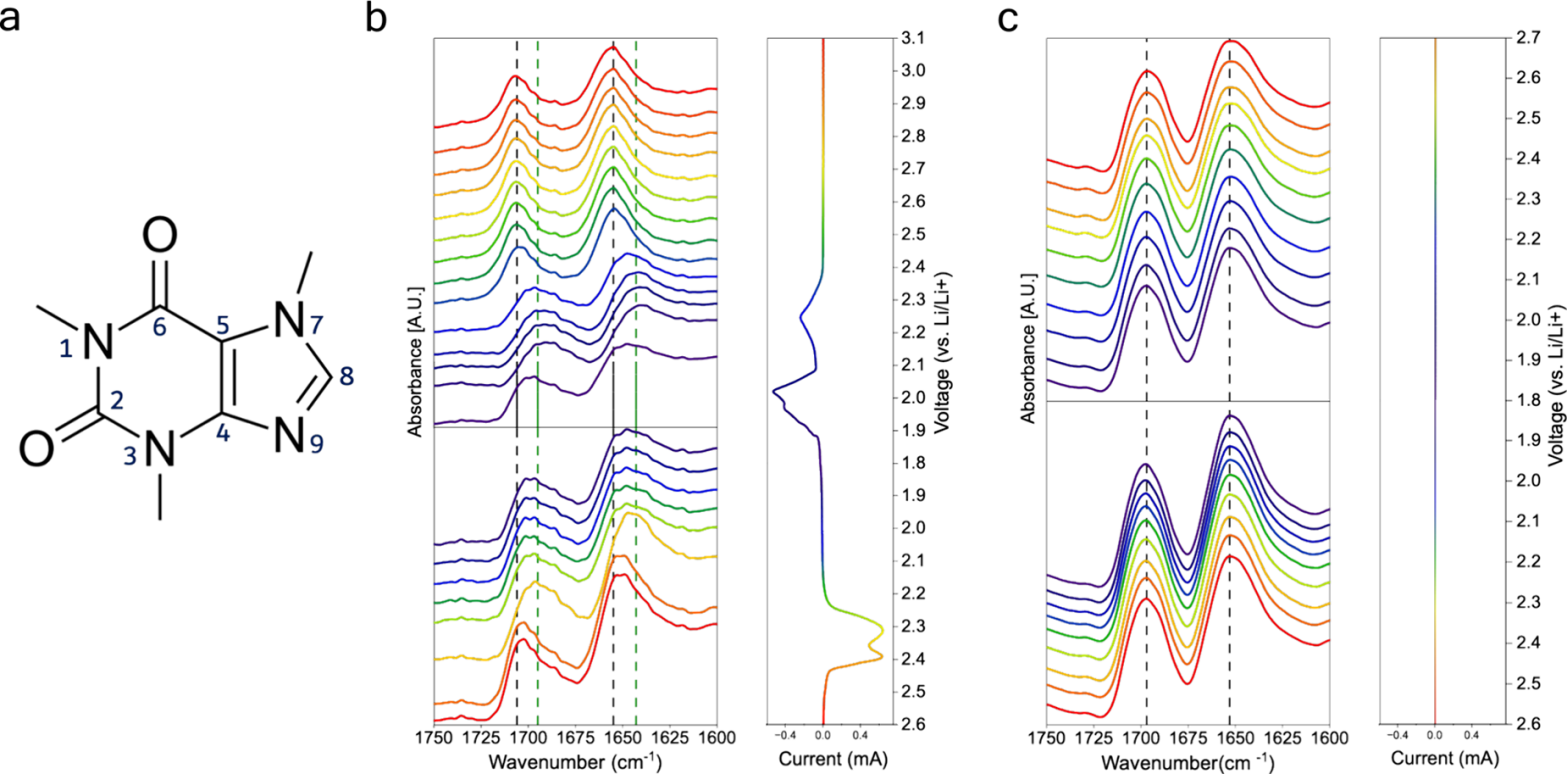
(a) Numbered caffeine molecule. (b) FTIR spectra of an *in-operando* lithium–sulfur coin cell with a cathode containing sulfur and caffeine with spectra corresponding to discharging and charging sweeps of the CV. Two dashed lines highlight peak shifting. (c) FTIR spectra of an *in-operando* coin cell with a cathode that has caffeine but no sulfur. Dashed lines show no peak shifting.

Figure 3b illustrates the changes in FTIR spectra of a 4:1 cathode during the first *in-operando* CV cycle (the second and third are shown in Figure S9). In the top half of the figure, spectra lines from the top (red) to bottom (violet) represent the cathode state as the battery progresses through its discharge. In the bottom half, the spectra lines from top (violet) to bottom (red) represent the cathode state as the battery charges. To the right of the figure, is the vertical representation of the corresponding CV performed on the *in-operando* cell. The CV curve is color coded, so each spectrum matches its corresponding voltage location. As shown, when the battery is discharged from 2.6 to 1.8 V, the C6 carbonyl peak shifts from 1,655 to 1,642 cm^–1^. The C2 carbonyl peak also shifts from 1,707 to 1,696 cm^–1^. Figure 3c represents an identical cell prepared without any sulfur added, exhibiting a carbonyl stretching region that is unperturbed, irrespective of the applied voltage. Therefore, we assign the origin of the spectral shifts to a polar interaction between caffeine and the lithium polysulfides.

Furthermore, the peak shifting phenomenon correlates strongly with certain voltages during discharge and charge. Again, in Figure 3b, both carbonyl peaks initially start to shift at around 2.35 V. This voltage corresponds to the onset of long chain lithium polysulfide formation,^41^ illustrating that the shift occurs only once polysulfides are present. Upon analyzing the S–S region of the *in-operando* FTIR data, we also observe polysulfide formation that corresponds precisely with this voltage and carbonyl peak shift (Figure S10). Interestingly, the way the two carbonyl peaks shift is slightly different. The C6 carbonyl peak first shifts by 8 cm^–1^ between 2.35 and 2.1 V. In contrast, the C2 carbonyl peak shifts by 11 cm^–1^ in this voltage window, which is the full magnitude of its shift during this discharge. Continuing the discharge from 2.1 V, long chain polysulfides are further reduced.^41^ From 2.1 to 1.9 V, the C6 carbonyl peak shifts a further 5 cm^–1^. Meanwhile, the C2 carbonyl peak remains fixed at 1,696 cm^–1^. This behavior is demonstrated again in subsequent cycles. For both the C2 and C6 carbonyls, the larger initial peak shift beginning at 2.35 V could be because there are more unoccupied sites on the caffeine molecules. As the polysulfide reduction continues, the available sites likely diminish. The rapid initial wavenumber shift may also be assisted by the absence of long side chains on caffeine. If long side chains existed, they might sterically hinder the polysulfide adsorption.^17^

We hypothesize that the difference in peak shifting behavior may be due to the difference in the polarity of the two carbonyl groups on caffeine. In a study performed by De Taeye et al.,^42^ they showed that the carbonyl groups were the preferred polar binding sites on caffeine by calculating formation constants and enthalpies of formation. A computational study also showed the carbonyl group as the preferred interaction site.^43^ Additionally, De Taeye et al. determined that the C2 carbonyl group had a greater ionization potential than the C6 carbonyl, making it the more likely location for polar interactions between the two carbonyls. On the basis of the strong polar interaction potential, we speculate that the carbonyl groups on caffeine could be interacting with the lithium of the polysulfides to bind them via a polar–polar interaction, thereby hindering their mobility. We also see a more rapid shift at the C2 carbonyl site and a more gradual shift at the C6 carbonyl site. This could mean that the C2 carbonyl site interacts more strongly with the lithium polysulfides, agreeing with the trends from the previous studies.^42,43^ As the discharge prolongs, the C6 carbonyl site has more time to bind polysulfides, which could explain why it exhibits an ultimate wavenumber shift similar to that of the C2 carbonyl.

As the discharge concludes at voltages of 1.9–1.8 V, the wavenumber of both the C2 and C6 carbonyl peaks returns to near its original value. This behavior suggests that lithium polysulfides are released from caffeine as they are reduced to Li_2_S. The bottom portion of Figure 3b shows that the carbonyl peak shifting phenomenon also occurs during the charging phase of the CV. As the cell is charged, redox activity beginning around 2.3 V indicates that Li_2_S is oxidizing back into lithium polysulfides. Corresponding with this voltage, the carbonyl peaks again show a large shift, which further supports the idea that caffeine binds lithium polysulfides. As the charging completes and polysulfides become elemental sulfur again, the carbonyl peaks demonstrate a reversal to near their original peak positions. The carbonyl peaks shifting back to near their original value suggests that caffeine molecules are not permanently binding many polysulfides during charging and discharging.

In this study, we performed 3 consecutive cycles on the *in-operando* FTIR cell with corresponding CV data (Figure 4). The difference in the C6 and C2 carbonyl peak shifting behaviors during discharge can be seen. The precise magnitude of the carbonyl peak shifts for each cycle can be found in Table S2. After the first cycle, we observe a hysteresis in the carbonyl peak wavenumbers, and in the third cycle, there appears to be an unrecovered drop in the C2 carbonyl peak. This is because we are tracking the middle position of the carbonyl peaks that becomes broadened. We hypothesize that the broadening may be due to the emergence of multiple caffeine species, causing the peak to span a larger wavenumber range (Figure S11). Prior to the onset of cycling, the liquid electrolyte can solvate a majority of the caffeine additive. Therefore, we initially observed single carbonyl peak positions. However, as cycling commences, the liquid electrolyte begins to deplete due to SEI formation.^44,45^ SEI reactions that persist throughout cycling may lower the amount of caffeine that can remain solvated, and thus we see the emergence of a solid and likely microcrystalline phase of caffeine. Furthermore, it has been found that, after the initial discharge, lithium polysulfides remain in the electrolyte, even when the battery is charged.^41^ This could lead to a third species of caffeine that is likely due to an interaction with these remaining polysulfides. Since these three populations of caffeine appear in similar locations in the IR spectrum, it would manifest as a peak broadening effect. Nevertheless, upon close inspection of the spectra, we observe the reversal of peak shifting in each cycle.

**Figure 4:**
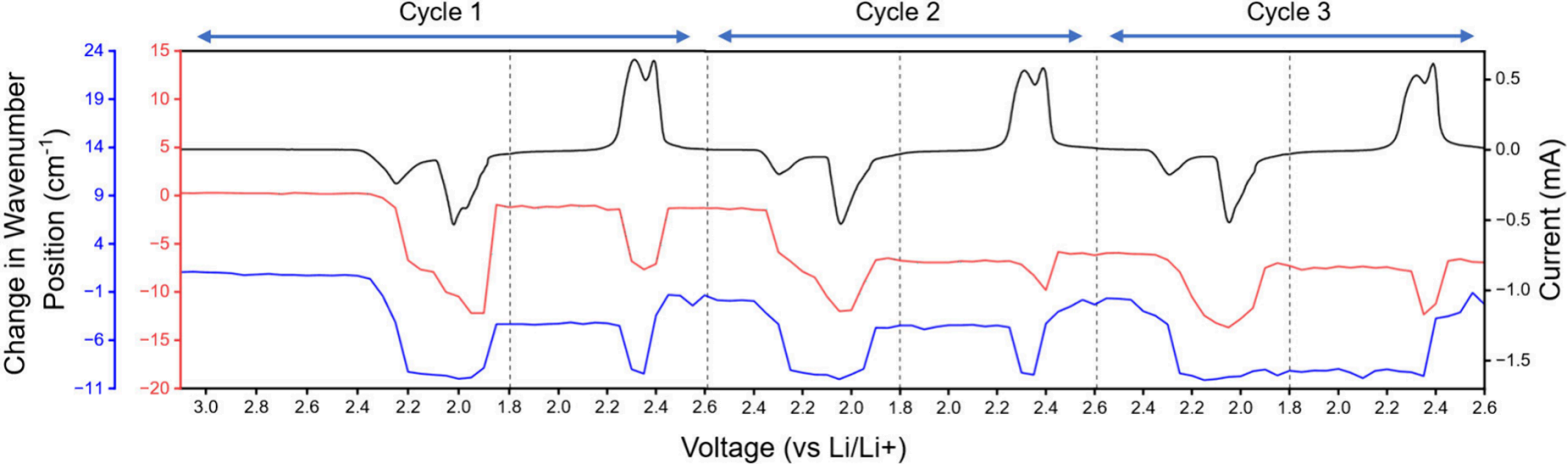
Wavenumber shifting associated with the C2 (blue) and C6 (red) carbonyl peaks, corresponding to the voltage and current during *in-operando* CV sweeps (black).

In this work, caffeine was studied as a novel cathode additive for Li–S batteries. Shuttle current measurements showed that the incorporation of caffeine into the cathode reduced the polysulfide concentrations in the electrolyte. Furthermore, we observed a 40% improvement in the galvanostatic cycling capacity at 300 cycles. To fundamentally investigate the lithium polysulfide interaction with caffeine, we used *in-operando* FTIR. A significant shift in the caffeine carbonyl peak positions indicated that caffeine could reversibly bind lithium polysulfides as the battery was discharged and charged. This shift correlated strongly with the 2.3 V discharge plateau of the *in-operando* CV, suggesting that caffeine rapidly binds these species as they are formed in the battery. After analyzing the different carbonyl peak shifting behaviors, we proposed that the C2 carbonyl was the preferred binding site between the two carbonyl groups. Further optimization of the cathode fabrication process to increase the caffeine loading without fracturing the slurry and exploration of other caffeine incorporation methods could see improvements in the distribution and amount of caffeine that can be added effectively to the cathode for heightened performance. Finally, this work serves as a fundamental study that identifies the likely interaction between caffeine molecules and lithium polysulfides via carbonyl groups and demonstrates its efficacy in a Li–S battery.

## Supporting Information Available

Experimental materials and methods and additional experiments and analysis (PDF)
