# Supplementary material for: In-Operando FTIR Spectroscopy Study on Rapid Polysulfide Binding via Caffeine in Lithium–Sulfur Batteries
Source: J Phys Chem Lett. 2024 Dec 2;15(49):12164–70. doi: 10.1021/acs.jpclett.4c02789 (PMC11648080; doi:10.1021/acs.jpclett.4c02789)
Supplement: Supplementary file 1 — jz4c02789_si_001.pdf [file jz4c02789_si_001.pdf]

Supporting Information for:

In-Operando FTIR Spectroscopy Study on Rapid Polysulfide Binding via Caffeine in Lithium-Sulfur Batteries

Taber Yim[1,2‡], Rhyz Pereira[1,2‡], Jantakan Nedsaengtip[2], Aaron T. Fafarman[1], and Vibha Kalra[1,2]*

‡These authors contributed equally.

1 Department of Chemical and Biological Engineering, Drexel University, Philadelphia, PA 19104, United States

2 Smith School of Chemical and Biomolecular Engineering, Cornell University, Ithaca, New York 14853, United States

Corresponding Author: Vibha Kalra - vk69@cornell.edu

Experimental Materials and Methods

Materials

Caffeine and poly(vinylidene fluoride) (PVDF) were purchased from Sigma Aldrich. Sulfur (99.5%, sublimed, 100 mesh), lithium (250 μm thick and 15.6 mm diameter), and carbon black Super P Conductive (99+% metals basis) were purchased from Alfa Aesar. N-methyl-2-pyrrolidone (NMP extra dry, 99.5%) was purchased from Thermo Scientific. Commercial ether electrolyte (Dimethoxyethane:1,3 Dioxolane 1:1 by volume with 1M lithium bis(trifluoromethanesulfonyl)imide and 1 wt% lithium nitrate (DME:DOL 1:1 vol% with 1M LiTFSI and 1 wt% $LiNO_3$)) was purchased from Gotion. Trilayer commercial battery separators (Celgard 2325) were purchased from Celgard.

Cathode fabrication

Cathodes for Li-S cells were produced using a slurry casting method. Conventional cathodes with no caffeine had a composition of 50 wt% sulfur, 40 wt% carbon, and 10 wt% PVDF. When incorporating caffeine, all aforementioned material masses, including sulfur, were kept constant with respect to the conventional cathode. The mass of caffeine was added with respect to the mass of sulfur in ratios of 10:1, 4:1, and 2:1 (sulfur:caffeine). Hereafter, cathodes with caffeine in them will be termed by their ratio (for example a cathode with a 4:1 ratio of sulfur to caffeine in it will be called a 4:1 cathode or 4:1 cell). To create the cathode, carbon black and sulfur were first mixed as dry powders in a planetary mixer (Thinky ARM-310). When being used, caffeine was also mixed as a dry powder in the mixer prior to its further mixing with dry carbon and sulfur powders. Separately, PVDF was dissolved in NMP. Once dissolved, the PVDF solution was added to the dry powder mixture and mixed in the planetary mixer. NMP was added until the slurry viscosity was appropriate for blade casting on aluminum foil. Once casted, the slurry was dried at 40 °C for 6 hours under vacuum followed by a further 12 hours under vacuum without heat. 11 mm diameter circular cathodes were punched from the dried slurry. The average areal sulfur loading for these cathodes was 1.5 mg S cm^{-2}. Cathodes for *in-operando* FTIR Li-S cells were produced by casting the above detailed cathode slurry mixture directly onto a Celgard film and then dried in the same manner. The sulfur loading of these cathodes was the same.

Electrochemical Characterization

Cathodes were transferred to an argon-filled glovebox (MBraun LABstar, O_2 and H_2O levels <1 ppm). The Li-S coin cells were fabricated using CR2032 casings, circular lithium anodes, a Celgard 2325 separator, and Gotion commercial ether electrolyte. The electrolyte was added at an E/S ratio of 20 (E = microliters of electrolyte, S = milligrams of sulfur). The assembled coin cells were rested at room temperature and open circuit voltage for 12 hours before initiating electrochemical experiments. Galvanostatic cycling tests were performed on a battery cycler (Neware BTS 4000) with a cycling profile of 2 cycles at C/10, 2 cycles at C/5, followed by extended cycling at C/2 (1C = 1,672 mAh g^{-1}_{Sulfur}) between 1.8 and 2.6 V. Cyclic voltammetry (CV) was performed on a potentiostat (Biologic VMP3) at a scan rate of 0.5 mV s^{-1} between 1.8 V and 2.6 V with respect to Li/Li+. Polysulfide shuttle current tests were performed by resting cells for 2 hours at open cell voltage (OCV), performing 2 C/10 cycles, charging them to 2.6 V, allowing them to rest for 10 minutes to stabilize at an OCV, then holding that OCV with chronoamperometry for 4 hours to measure the current response. Electrochemical impedance spectroscopy was conducted on the Biologic potentiostat from 1 MHz to 100 mHz with a 10 mV potential amplitude. Li_2S nucleation tests were performed by discharging a Li-S coin cell galvanostatically at a C/10 current rate from OCV to 2.1V, then using chronoamperometry to hold the cell at 1.95 V for 3 hours and measuring the current response.

Material Characterization

The morphology of the cathodes was observed using a scanning electron microscope (SEM, Thermo-Fisher Apreo 2S) with a 10 mm working distance. Elemental mapping was achieved using energy dispersive X-ray spectroscopy (EDS, ChemiSEM Technology, Thermo-Fisher). Material bond signatures were collected with a Fourier transform infrared spectrometer (FTIR, Nicolet iS50, Thermo-Fisher) using an extended range, attenuated total reflection (ATR) diamond. UV-vis spectroscopy was performed to measure polysulfide solution intensity (Cary 5000). X-ray photoelectron spectroscopy was performed to study the cathode surface chemical environment (XPS, Thermo Scientific Nexsa G2).

In-operando Characterization

Our lab has previously designed an *in-operando* FTIR cell with two important and unique features.[1, 2] The first feature is using an ATR accessory on a stainless-steel puck with aluminum contacts. This acts as the positive current collector, replacing the conventional bottom CR2032 coin cell cap. The second feature is a freestanding cathode slurry coated on a Celgard separator. This eliminates the need for the conventional aluminum current collector that would otherwise block the IR signal.

Coin cells for *in-operando* FTIR were also assembled in the argon-filled glovebox (O_2 and H_2O levels <1 ppm). They were assembled with the slurry casted on Celgard cathode, a Celgard 2325 separator, lithium anode, and Gotion commercial ether electrolyte. These cells had an E/S ratio of 30 to mitigate drying out of components in the coin cell while cycling. FTIR spectra (FTIR-ATR, Nicolet iS50, Thermo-Fisher) and CV (Biologic VMP3) were collected simultaneously. CV was performed at a scan rate of 0.02 mV s^{-1} between 1.8 V and 2.6 V.

Additional Data Analysis

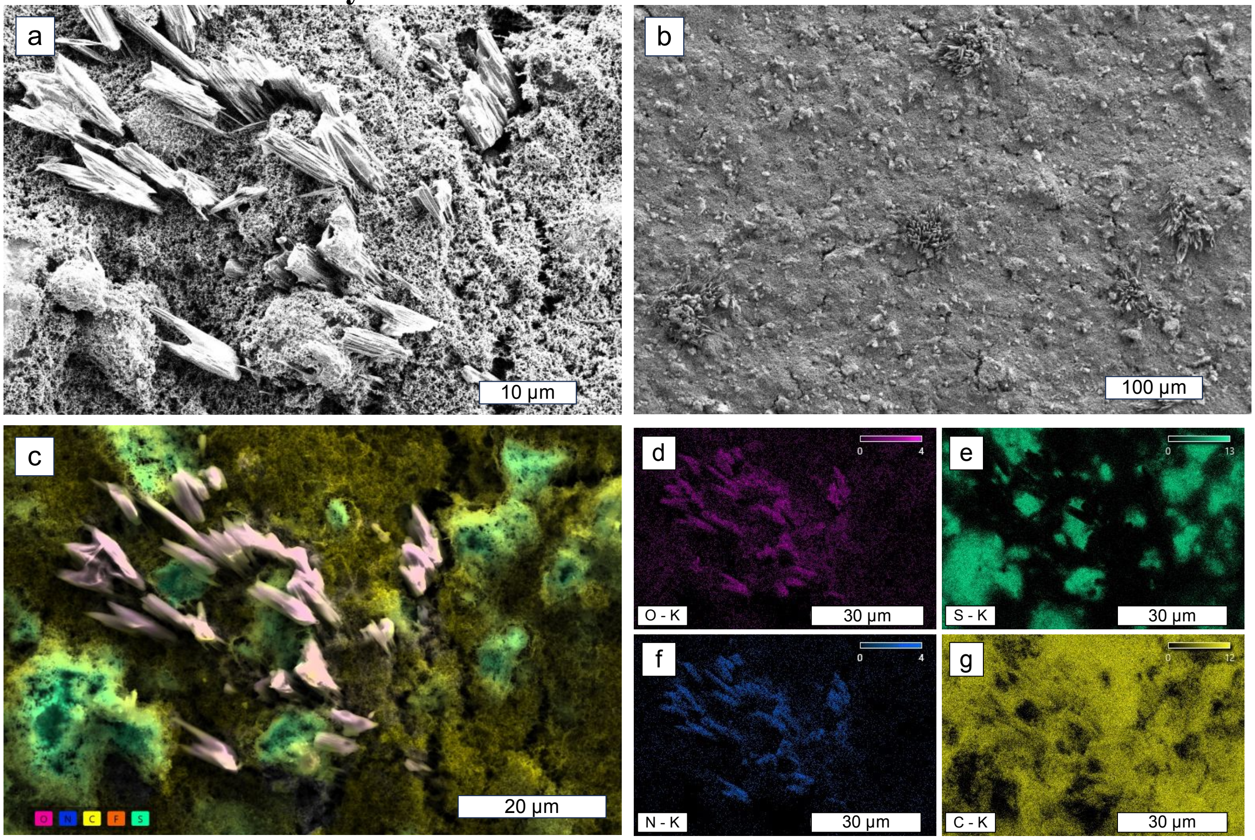

Figure S1. SEM images of caffeine incorporated into a carbon-sulfur slurry (a) at 2,000x magnification and (b) 200x magnification. (c) SEM image of the region shown in (a) at 1,500x magnification with EDS elemental mapping overlaid to highlight the presence of caffeine in the cathode with individual elemental maps of (d) oxygen, (e) sulfur, (f) nitrogen, and (g) carbon.

Figure S1a shows an SEM image of caffeine crystals incorporated into a conventional carbon and sulfur cathode slurry. Distinct crystal structures can be seen protruding from the cathode surface. It illustrates how the caffeine powder initially used in the cathode slurry formulation exhibits a self-assembling behavior during the cathode fabrication process. **Figure S1b** shows the same region at a lower magnification and reveals that the crystal structures tend to form in clusters on the cathode surface. These clusters are uniformly dispersed across the cathode surface. **Figure S1c** shows the SEM image from **Figure S1a** with an EDS elemental map overlaid. The caffeine crystal near the center of the image is highlighted in pink and some blue because it has oxygen (pink) and nitrogen (blue) atoms from its carbonyl and tertiary amine groups, respectively. The surrounding material shows sulfur in a light green color and carbon as yellow. **Figures S1d-g** respectively show the individual elemental maps of oxygen, sulfur, nitrogen, and carbon with the colors described.

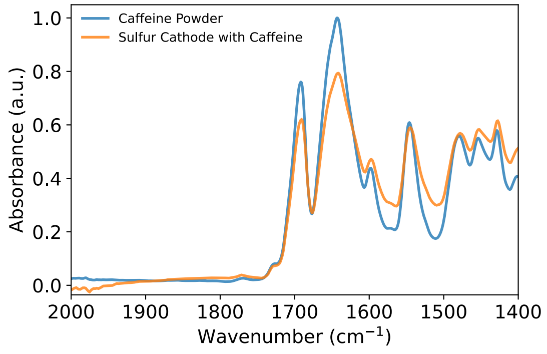

Figure S2. Spectra comparing caffeine powder to a 4:1 cathode with caffeine incorporated showing identical peaks in the range of 1,400 and 1,800 cm^{-1}.

Figure S2 shows that a sulfur cathode with caffeine incorporated into it still exhibits the distinct caffeine carbonyl peaks after the cathode processing. This demonstrates that the carbonyl groups of caffeine are unchanged by the cathode manufacturing process and there are no other components with interfering signals.

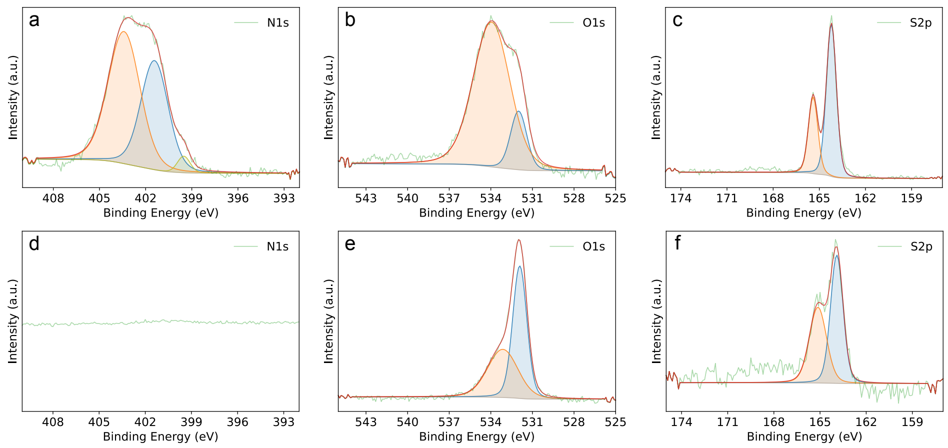

Figure S3. X-ray photoelectron spectroscopy spectra of a cathode with caffeine (a) N1s (b) O1s (c) S2p and a cathode without caffeine (d) N1s (e) O1s (f) S2p.

Figure S3 shows that the XPS spectra for the cathode with caffeine (Figure S3a) shows the peaks related to the imidazole group (401.4 eV and 403 eV) and amine groups (399.5 eV) of caffeine in the N1s whereas the cathode without caffeine shows no signal (Figure S3d). In the O1s spectra for the cathode with caffeine, there is a strong signal assigned to the carbonyl group at 533.6 eV (Figure S3b). In the spectra for the cathode without caffeine (Figure S3e) the peak intensity for the carbonyl group is much lower since this signal comes only from the carbon black and no additional caffeine. Furthermore, the S2p spectra for both the cathode with and without caffeine remain alike (Figure S3c, S3f), showing that there is no effect on the active material.

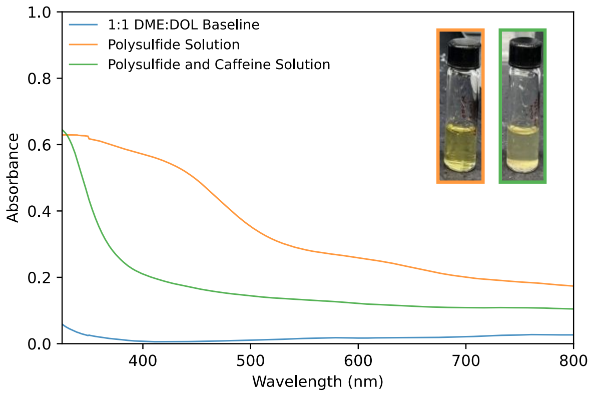

Figure S4. UV-vis spectra and corresponding photographs for polysulfide solution with and without exposure to caffeine powder.

Figure S4 shows UV-vis spectra of a 2 mM solution of Li_2S_4 lithium polysulfide solution before and after exposure to caffeine powder. Polysulfide solution was made by vigorously mixing stoichiometric amounts of elemental sulfur (S_8) and lithium sulfide (Li_2S) in a solution of DME:DOL (1:1 v/v). When exposed to caffeine, the solution becomes clearer and there is a corresponding reduction in intensity for the polysulfide peak in the UV-vis spectra at around 420 nm.[3] This supports our claim that caffeine can adsorb polysulfides.

Table S2. Comparison of various published works focused on polysulfide shuttle inhibition

Reference	Capacity Retention (mAh g^{-1})	Cycle Number	C-Rate
[4]	675	400	1.0
[5]	792	250	0.5
[6]	581	200	0.5
[7]	575	150	0.5
[8]	509	500	0.5
This work	500	300	0.5
[9]	471	500	0.5
[10]	446	500	0.5
[11]	522	200	0.2
[12]	850	50	0.1

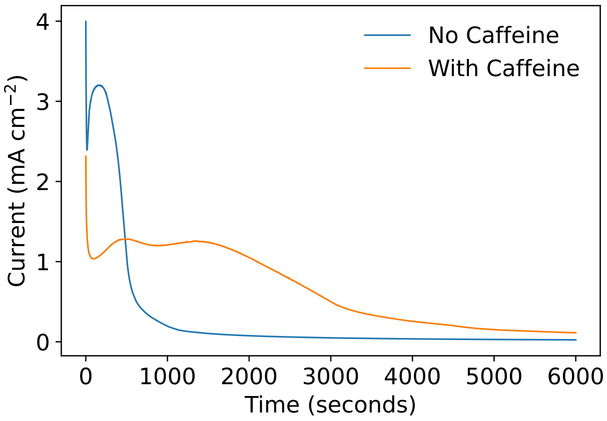

Figure S5. Li_2S nucleation measurements comparing a cathode without caffeine (blue) and a cathode with caffeine (orange).

Figure S5 shows that in the cell without caffeine, the peak current achieved is higher than in the cell with caffeine. Although, the duration of nucleation is longer when caffeine is present. This illustrates that more capacity is achieved in the cell with caffeine, but nucleation is achieved more easily in the cell without caffeine. This supports the idea that Li_2S may deposit in localized areas in the cathode when caffeine is present, creating slightly more resistance to deposition.

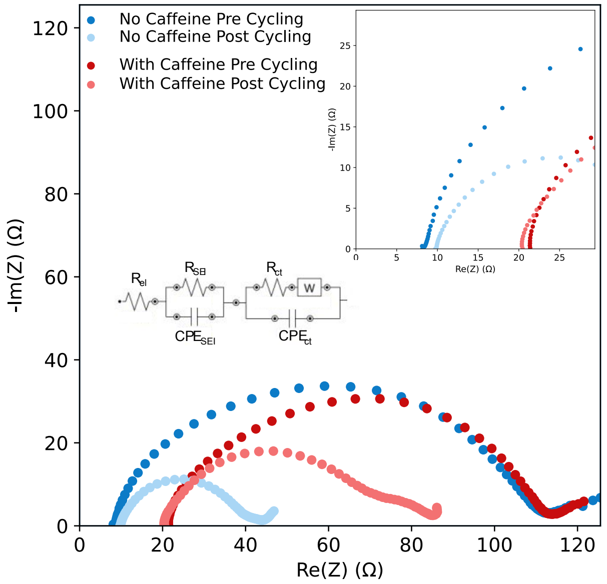

Figure S6. EIS spectra showing the resistance in coin cells with and without caffeine.

The EIS curves for both cells with and without caffeine have a similar resistance before cycling. After cycling, the resistance in both cells decreases due to the rearrangement of sulfur. In the cell with caffeine, there is a slightly larger charge transfer resistance. This is to be expected, as the caffeine molecules are binding polysulfides. Still, the overall benefit of increased capacity in the cell is an advantage.

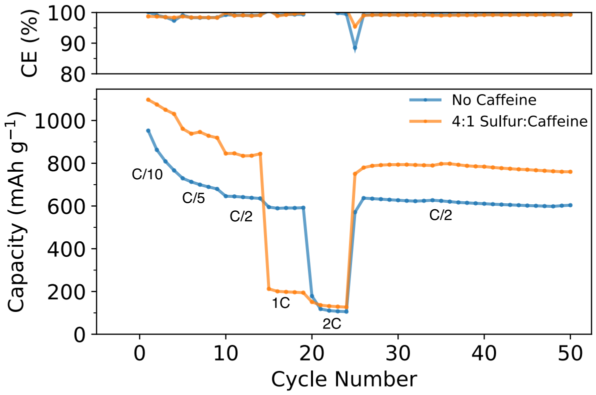

Figure S7. Rate capability test for coin cells with and without caffeine.

We performed 5 cycles each at C-rates of C/10, C/5, C/2, 1C, and 2C. The cell with caffeine performed better than the cell without caffeine at the lower C-rates (C/10, C/5, C/2). However, the cell with caffeine did not perform as well at 1C and 2C. The cell without caffeine was able to cycle at 1C, although both cells were unable to perform well at 2C. Both cells recovered after returning to the C/2 rate. The faster C-rates require faster deposition and reduction processes which is more limited in the cell with caffeine. These results support our discussion about the voltage curves and the increased overpotential at 2.1 V in the manuscript (**Figure 2**).

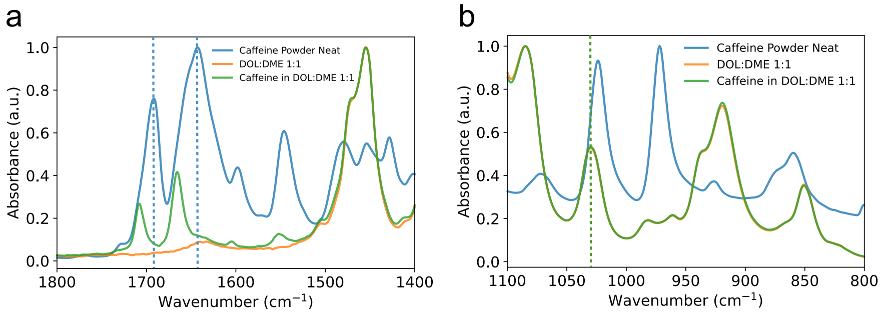

Figure S8. (a) FTIR spectra of caffeine powder and caffeine powder dissolved in DME:DOL 1:1 ether solvents in the region of 1400-1800 wavenumber and (b) in the region of 800-1100 wavenumber.

Figure S8 shows the characteristic peaks from caffeine in blue. There is no interference from the ether solvent in the carbonyl region.

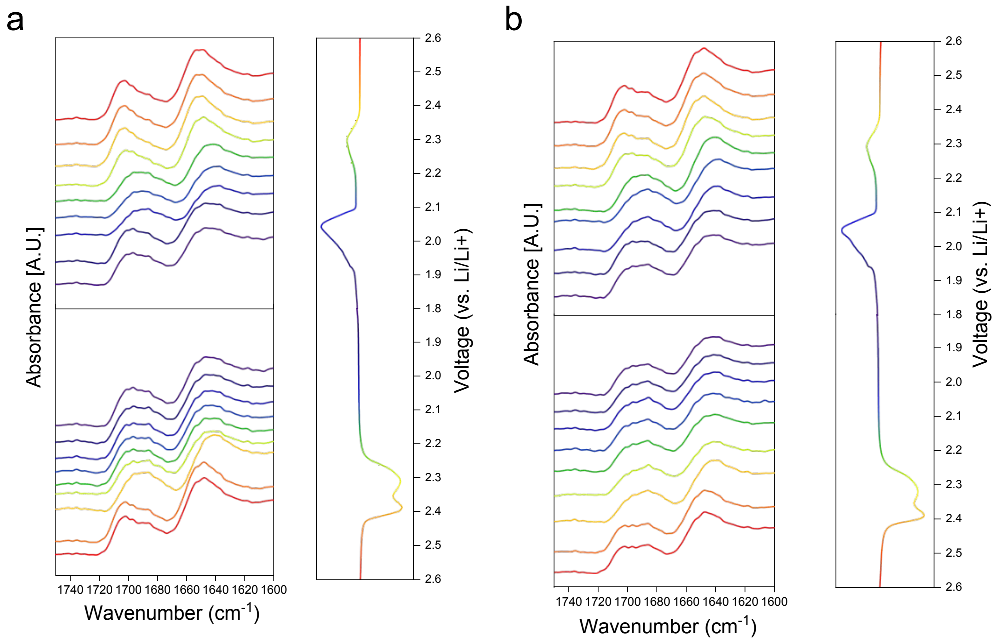

Figure S9. In-operando FTIR spectra of a lithium-sulfur battery with caffeine as a cathode additive showing caffeine carbonyl peaks shifting during discharge and charge (a) second cycle and (b) third cycle.

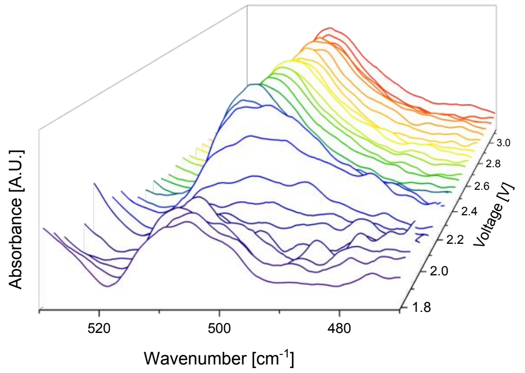

Figure S10. In-operando IR spectra showing polysulfide evolution during the first discharge of the in-operando CVs.

In this work, we observed the presence of polysulfides by the appearance of peaks in the S-S IR region (**Figure S10**). The peak at 510 cm^{-1} is the C-F vibration from the electrolyte salt and is prominent during the OCV resting phase.[1] As elemental sulfur begins to reduce, long chain polysulfides appear and are visible as a peak around 502 cm^{-1}. This peak diminishes as the long chain polysulfides are reduced to shorter chain polysulfides and other peaks begin to appear at lower wavenumbers (500 cm^{-1} to 475 cm^{-1}).[13] This behavior is consistent with our earlier studies on polysulfide evolution using *in-operando* FTIR.[1] These peaks also correspond to the carbonyl peak shifting phenomenon where we observe the carbonyl peak shift at voltages when the polysulfides begin to appear.

Table S2. Difference in carbonyl peaks wavenumber as a result of the shifting behavior exhibited during each cycle.

		Wavenumber Shift (Δ#)											
		Discharge 1		Charge 1		Discharge 2		Charge 2		Discharge 3		Charge 3	
		High to Low	Low to High	High to Low	Low to High	High to Low	Low to High	High to Low	Low to High	High to Low	Low to High	High to Low	Low to High
Carbonyl Peak	~1,655 cm^{-1}	12.5	10.99	6.44	6.37	10.67	5.26	3.07	3.85	7.5	6.41	5.06	5.4
	~1,700 cm^{-1}	11.16	5.67	5.15	8.16	8.69	5.59	5.12	7.78	8.49	0.96	0.54	8.64

Table S1 shows the total change in peak wavenumber for each carbonyl peak. The C6 carbonyl peak corresponds to the 1,655 cm^{-1} location and the C2 carbonyl peak corresponds to the 1,700 cm^{-1} location. Each half of a given cycle has two parts, discharge and charge. The three *in-operando* cycles are each delineated into these two distinct parts. Furthermore, each charge and discharge exhibited a shift from a higher wavenumber value to a lower wavenumber value (denoted as “High to Low”) and then a reversal from the lower wavenumber to a higher wavenumber (denoted as “Low to High”). The magnitude of these shifts is indicated by the value listed in the box.

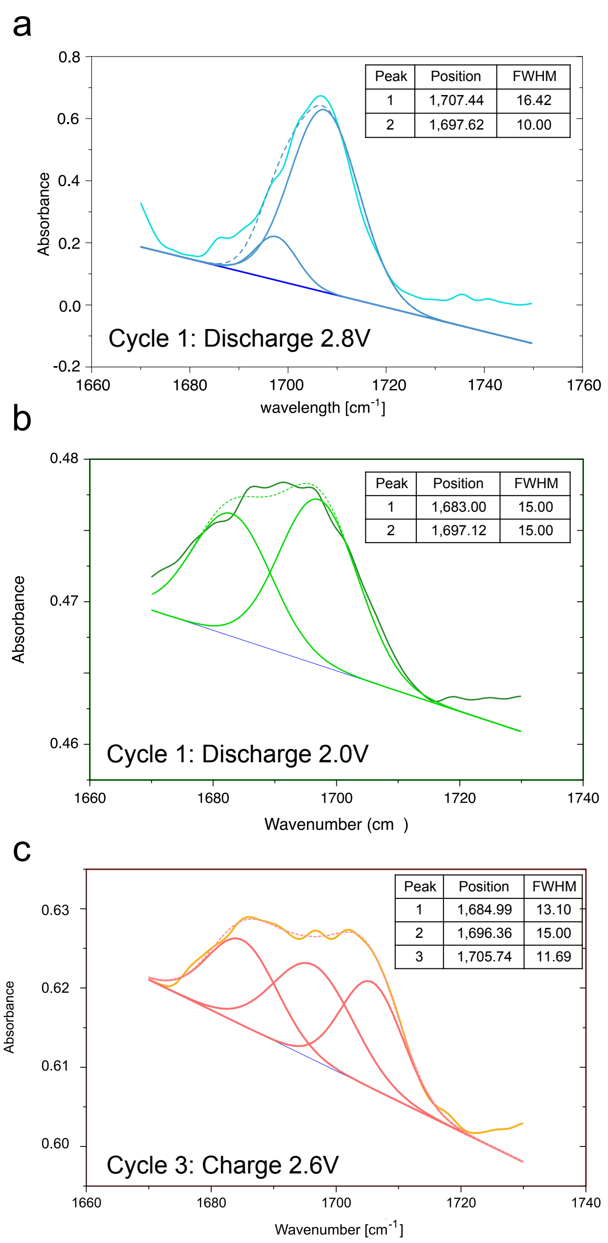

Figure S11. Fitted Gaussian curves to spectra from various points during in-operando cycling at (a) 2.8V during the first discharge (b) 2.0 V during the first discharge and (c) 2.6 V during the third charge.

Fitted Gaussian curves show the presence of 3 distinct species of caffeine emerging by the third cycle. At 1707 cm^{-1} the solvated caffeine species exists. This is the dominant species before cycling begins. At 1,697 cm^{-1} the solid micro crystalline phase of caffeine exists. This phase becomes apparent after a few cycles. At 1,684 cm^{-1} the caffeine species that is interacted with polysulfides exists. This appears distinctly during the discharge of the first cycle and then persist even in the charged state, since polysulfide species remain after their formation.

Citations

(1) Dillard, C.; Singh, A.; Kalra, V. Polysulfide Speciation and Electrolyte Interactions in Lithium–Sulfur Batteries with in Situ Infrared Spectroelectrochemistry. *J. Phys. Chem. C* **2018**, *122* (32), 18195-18203. DOI: 10.1021/acs.jpcc.8b02506.
(2) Pereira, R.; Sarode, K. K.; Rafie, A.; Fafarman, A.; Kalra, V. In-Operando FTIR Study on the Redox Behavior of Sulfurized Polyacrylonitrile as Cathode Material for Li–S Batteries. *J. Phys. Chem. C* **2023**, *127* (39), 19356-19365. DOI: 10.1021/acs.jpcc.3c03421.
(3) He, Q.; Freiberg, A. T. S.; Patel, M. U. M.; Qian, S.; Gasteiger, H. A. Operando Identification of Liquid Intermediates in Lithium–Sulfur Batteries via Transmission UV–vis Spectroscopy. *J. Electrochem. Soc.* **2020**, *167* (8). DOI: 10.1149/1945-7111/ab8645.
(4) Liu, Y.; Ma, Z.; Yang, G.; Wu, Z.; Li, Y.; Gu, J.; Gautam, J.; Gong, X.; Chishti, A. N.; Duan, S.; et al. Multifunctional ZnCo2O4 Quantum Dots Encapsulated In Carbon Carrier for Anchoring/Catalyzing Polysulfides and Self-Repairing Lithium Metal Anode in Lithium-Sulfur Batteries. *Advanced Functional Materials* **2021**, *32* (12). DOI: 10.1002/adfm.202109462.
(5) Shi, Y.; Li, D.; Sun, X.; Xue, Y.; Li, Z.; Fu, Y.; Luo, C.; Lin, Q.; Gui, X.; Xu, K. Cationic Polymer Binder for Simultaneously Propelling Ion Transfer and Promoting Polysulfide Conversion in Lithium–Sulfur Batteries. *ACS Applied Polymer Materials* **2024**, *6* (13), 7430-7440. DOI: 10.1021/acsapm.4c00668.
(6) Yang, Y.; Qiu, J.; Cai, L.; Liu, C.; Wu, S.; Wei, X.; Luo, D.; Zhang, B.; Yang, X.; Hui, K. N.; et al. Water-Soluble Trifunctional Binder for Sulfur Cathodes for Lithium-Sulfur Battery. *ACS Appl Mater Interfaces* **2021**, *13* (28), 33066-33074. DOI: 10.1021/acsami.1c07901.
(7) Chang, C.-H.; Chung, S.-H.; Han, P.; Manthiram, A. Oligoanilines as a suppressor of polysulfide shuttling in lithium–sulfur batteries. *Materials Horizons* **2017**, *4* (5), 908-914. DOI: 10.1039/c7mh00510e.
(8) Wu, T.; Ye, J.; Li, T.; Liu, Y.; Jia, L.; Sun, L.; Liu, J.; Xie, H. Tetrathiafulvalene as a multifunctional electrolyte additive for simultaneous interface amelioration, electron conduction, and polysulfide redox regulation in lithium-sulfur batteries. *J. Power Sources* **2022**, *536*. DOI: 10.1016/j.jpowsour.2022.231482.
(9) Liu, M.; Chen, X.; Chen, C.; Ma, T.; Huang, T.; Yu, A. Dithiothreitol as a promising electrolyte additive to suppress the "shuttle effect" by slicing the disulfide bonds of polysulfides in lithium-sulfur batteries. *J. Power Sources* **2019**, *424*, 254-260. DOI: 10.1016/j.jpowsour.2019.03.113.
(10) Xie, F.; Xu, C.; Liang, Y.; Tian, Z.; Ma, C.; Xu, S.; Li, Z.; Rehman, Z. U.; Yao, S. Cubic FeS2 enabling robust polysulfide adsorption and catalysis in lithium/sulfur batteries. *Journal of Energy Storage* **2023**, *72*. DOI: 10.1016/j.est.2023.108712.
(11) Han, P.; Chung, S. H.; Chang, C. H.; Manthiram, A. Bifunctional Binder with Nucleophilic Lithium Polysulfide Immobilization Ability for High-Loading, High-Thickness Cathodes in Lithium-Sulfur Batteries. *ACS Appl Mater Interfaces* **2019**, *11* (19), 17393-17399. DOI: 10.1021/acsami.9b02399.
(12) Castillo, J.; Santiago, A.; Judez, X.; Coca-Clemente, J. A.; Saenz de Buruaga, A.; Gomez-Urbano, J. L.; Gonzalez-Marcos, J. A.; Armand, M.; Li, C.; Carriazo, D. High Energy Density

Lithium-Sulfur Batteries Based on Carbonaceous Two-Dimensional Additive Cathodes. *ACS Appl Energy Mater* **2023**, *6* (6), 3579-3589. DOI: 10.1021/acsaem.3c00177.
(13) Rafie, A.; Pereira, R.; Shamsabadi, A. A.; Kalra, V. In Operando FTIR Study on the Effect of Sulfur Chain Length in Sulfur Copolymer-Based Li–S Batteries. *J. Phys. Chem. C* **2022**, *126* (30), 12327-12338. DOI: 10.1021/acs.jpcc.1c09124.
